# Designed Synthesis of Nanostructured Magnetic Hydroxyapatite Based Drug Nanocarrier for Anti-Cancer Drug Delivery toward the Treatment of Human Epidermoid Carcinoma

**DOI:** 10.3390/nano7060138

**Published:** 2017-06-06

**Authors:** Bharath Govindan, Beeseti Swarna Latha, Ponpandian Nagamony, Faheem Ahmed, Muheet Alam Saifi, Abdel Halim Harrath, Saleh Alwasel, Lamjed Mansour, Edreese H. Alsharaeh

**Affiliations:** 1 Department of Chemistry, Alfaisal University, P.O. Box 50927, Riyadh 11533, Saudi Arabia; fahahmed@alfaisal.edu; 2 Department of Nanoscience and Technology, Bharathiar University, Coimbatore 641046, India; ponpandian@buc.edu.in; 3 Department of Biotechnology, Indian Institute of Technology Madras, Chennai-600036, Tamil Nadu, India; swarnaiitm@gmail.com; 4 Department of Zoology, College of Science, King Saud University, P.O. Box 2455, Riyadh 11451, Saudi Arabia; muheetsaifi@gmail.com (M.A.S.); halim.harrath@gmail.com (A.H.H.); sawasel10@hotmail.com (S.A.); lamjed.mansour@gmail.com (L.M.)

**Keywords:** magnetic hydroxyapatite nanocomposites, cytotoxicity, human skin cancer, drug nanocarrier, andrographolide

## Abstract

Superparamagnetic Fe_3_O_4_ nanoparticles on hydroxyapatite nanorod based nanostructures (Fe_3_O_4_/HAp) were synthesized using hydrothermal techniques at 180 °C for 12 h and were used as drug delivery nanocarriers for cancer cell therapeutic applications. The synthesized Fe_3_O_4_/HAp nanocomposites were characterized by X-ray diffraction analysis (XRD), Field emission scanning electron microscopy (FESEM), Fourier transform infrared spectroscopy (FTIR), Brunauer-Emmett-Teller (BET)-analysis, and vibrating sample magnetometry (VSM). The morphologies of the Fe_3_O_4_/HAp nanocomposites show 15 nm Fe_3_O_4_ nanoparticles dispersed in the form of rods. The BET result shows that the synthesized samples have a high specific surface area of 80 m^2^ g^−1^ with mesoporous structures. Magnetic measurements revealed that the sample has high saturation magnetization of 18 emu/g with low coercivity. The Fe_3_O_4_/HAp nanocomposites had a large specific surface area (SSA), high mesoporous volume, and good magnetic property, which made it a suitable nanocarrier for targeted drug delivery systems. The chemotherapeutic agent, andrographolide, was used to investigate the drug delivery behavior of the Fe_3_O_4_/HAp nanocomposites. The human epidermoid skin cancer cells (A431) were used as the model targeting cell lines by treating with andrographolide loaded Fe_3_O_4_/HAp nanosystems and were further evaluated for their antiproliferative activities and the induction of apoptosis. Also, the present nanocomposite shows better biocompatibility, therefore it can be used as suitable drug vehicle for cancer therapy applications.

## 1. Introduction

Magnetic Fe_3_O_4_ nanoparticles (NPs) have gained much scientific attention due to their magnetic properties such as low coercivity, high saturation magnetization, and low Curie temperature [1,2]. Fe_3_O_4_ nanoparticles are being used in a sequence of advanced biomedical applications including magnetic resonance imaging (MRI), cell separation, hyperthermia, biosensing, and drug delivery for cancer therapy applications because of their advantages such as extremely low toxicity and good biocompatibility [3,4,5,6,7]. Therefore, Fe_3_O_4_ nanoparticles are considered to be the most favorable nanomaterials for biomedical applications. Various previous studies have reported that Fe_3_O_4_ nanoparticles were conjugated with the anti-cancer drug doxorubicin (DOX), and its ability as a model drug delivery platform was determined using HepG2 cells [6]. Moreover, thermo-sensitive molecules like azobis[*N*-(2-carboxyethyl)-2-methylpropionamidine] (Azo) were used as linker molecules between DOX and Fe_3_O_4_ nanoparticles for combined photothermal therapy and chemotherapy applications [7]. Under near infrared (NIR) light, the Fe_3_O_4_ NPs generate local heat, which prompts the decomposition of the Azo molecule and the release of DOX into cell lines to enhance the cytotoxic efficiency. However, it is more complicated and also affects normal cells. Recently, researchers have focused on pH dependent drug delivery platforms for cancer therapy and biomedical applications. Specifically, the nanomaterials should deliver anticancer drugs at the local pH (4.4) of cancer cells. On the other hand, hydroxyapatite (Ca_10_(PO_4_)_6_(OH)_2_, HAp) nanomaterials have become a topic of extreme interest due to their high loading capacity and drug delivery ability at pH 4.4 [8]. Basically, the geometry of HAp plays a key role in loading more anticancer drugs and effective drug delivery in the low pH environments of cancer cells.

The HAp crystal structure includes PO_4_^3−^, Ca^2+^, and OH^−^ ions, and the positive charges of Ca^2+^ sites are surrounded by the negative charges of tetrahedral PO_4_^3−^ units and OH^−^ ions and occupy columns parallel to the hexagonal axis [9]. The negative charges of PO_4_^3−^ and OH^−^ ions are present in the c-planes and the positive charges of Ca^2+^ ions are mainly present at the b-planes. These two different charges of HAp and their specific surface area (SSA) may be responsible for their higher adsorption of anticancer drugs. Also, the HAp was efficiently dissolved and releases maximum non-toxic calcium and phosphate ions at pH 4 to 5 [10,11]. Considering the abovestated advantages, the HAp is used as a potential candidate as a drug nanocarrier for cancer therapy applications. However, drug delivery into specific tissues and the rate of material recovery after drug delivery into cancer cells are limitations for drug delivery applications [12]. Recent investigations have shown that the combined effect of magnetic and hydroxyapatite nanostructures provides high drug loading capacity and an efficient drug delivery to specific targets with a controlled external magnetic field. Especially, the HAp/Fe_3_O_4_ nanocomposites are promising bio-nanomaterials for various applications like targeted drug delivery, orthopaedic, protein adsorption/pH dependent controlled release, hyperthermia-based anticancer treatments, and solid phase extraction of plasmid DNA [8,13,14,15]. Therefore, the synthesis of HAp/Fe_3_O_4_ nanocomposites is in high demand for biomedical drug delivery applications. 

Here in, superparamagnetic Fe_3_O_4_ and Fe_3_O_4_/HAp nanocomposites were prepared through a simple hydrothermal method at 180 °C for 12 h. The synthesized samples were characterized through various analytical techniques. The growth mechanism was presented based on the experimental results. The synthesized Fe_3_O_4_/HAp nanocomposites possess good magnetic property, high specific surface area (SSA), and high pore volume. These features make it a good biological potential candidate for higher drug loading capability and targeted drug release into specific cell lines. An anticancer drug loading and drug release of the Fe_3_O_4_/HAp nanocomposite were investigated using andrographolide as an anticancer drug against the human skin cancer cell line A431. Also, the synthesized Fe_3_O_4_/HAp nanocomposite shows excellent biocompatibility. Finally, it was concluded that the as-prepared Fe_3_O_4_/HAp nanocomposite shows high drug loading and releasing capability at a lower pH of 4.4, and is biocompatible. Hence, it can be used for drug delivery in cancer therapy applications.

## 2. Experiments

### 2.1. Materials

All the reagents, solvents, and chemicals used in the synthesis were of analytical or equivalent grade and were used as received without further purification. The di-ammonium hydrogen phosphate ((NH_4_)_2_(HPO_4_)), calcium chloride dihydrate (CaCl_2_·2H_2_O), Ferric trichloride hexahydrate (FeCl_3_·6H_2_O), Ferrous chloride tetrahydrate (FeCl_2_·4H_2_O), *N*-Cetyl-*N*,*N*,*N*-trimethyl ammonium bromide (CTAB), ammonium hydroxide (NH_4_OH), acetone, and ethanol were obtained from Sigma-Aldrich (Darmstadt, Germany). 

### 2.2. Synthesis of Fe_3_O_4_ Nanoparticles

In a typical synthesis procedure, 100 mM of FeCl_2_·4H_2_O and 200 mM of FeCl_3_·6H_2_O were dissolved in 25 mL of aqueous solution. Then, 10 mL of ammonium hydroxide (30%) was added into the above solution. This mixed solution was stirred vigorously for 30 min. Then the mixed solution was transferred into a Teflon lined stainless steel autoclave and maintained at 180 °C for 12 h. After cooling down to normal temperature, the black color solid product was separated by centrifugation for 5 min and washed with distilled water followed by ethanol three times. The final product was dried in a vacuum oven at 70 °C for 12 h for further characterization and applications. 

### 2.3. Synthesis of Fe_3_O_4_/HAp Nanocomposites

The Fe_3_O_4_/HAp nanocomposites were prepared through the hydrothermal method. In a typical procedure, 1 M of *N*-Cetyl-*N*,*N*,*N*-trimethyl ammonium bromide (CTAB) and 100 mM of (NH_4_)_2_ HPO_4_ were dissolved in 20 mL of double distilled water with constant stirring for 10 min. Also, 300 mM of CaCl_2_·2H_2_O was dissolved in 20 mL double distilled water with constant stirring for 10 min. Consequently, the CTAB-phosphate solution was mixed with a solution of calcium chloride and then the pH was adjusted to 10.5–11 by adding the ammonium hydroxide solution (30%) with constant stirring for 15 min. Then 10 mg of synthesized Fe_3_O_4_ nanoparticles were dispersed into the above solution and the translucent mixed solution was transferred to the Teflon-lined stainless steel autoclave at 180 °C for 12 h. After cooling down to normal temperature, the solid product was collected by centrifugation for 5 min and washed with distilled water followed by ethanol three times. Finally, the resulting Fe_3_O_4_/HAp nanocomposites were obtained after drying at 70 °C for 12 h under vacuum. 

### 2.4. Characterization

The XRD analysis of the synthesized samples was carried out at room temperature using a PAN analytical (X-Pert-Pro) diffractometer (Almelo, The Netherlands) with Cu Kα_1_ radiation (λ = 1.5406 Å) over a scanning interval (2θ) from 10° to 70°. The chemical composition and functional groups of the samples were studied using a Fourier transform infrared (FTIR) spectrometer (Bruker Tensor 27, Billerica, MA, USA), with a wavelength range of 4000–450 cm^−1^. The morphology of the synthesized samples was observed through field emission scanning electron microscopy (FESEM) (FEI Quanta-250 FEG, Hillsboro, OR, USA) coupled with energy dispersive X-ray analysis (9EDX) spectroscopy. The SSA and pore sizes were determined using BET-N_2_ adsorption-desorption experiments using a micromeritics ASAP 2020 surface area analyzer (Norcross, GA, USA).

### 2.5. In Vitro Cytotoxicity Studies

#### 2.5.1. Cell Culture Reagents 

Dulbecco’s Modified Eagles Medium (DMEM), Phosphate buffered saline (PBS), Fetal bovine serum (FBS), and penicillin were purchased from HIMEDIA, Mumbai, India. Dimethyl sulfoxide (DMSO), Neutral Red Powder, and Trypsin were purchased from MPBIO, Santa Ana, CA, USA. MTT (3-(4,5-dimethylthiazol-2-yl)-2,5-diphenyltetrazolium bromide) was obtained from BioBasic, Markham, ON, Canada.

#### 2.5.2. Cell Culture

The human skin cancer cell line A431 and normal human epidermal keratinocyte HaCaT cells were obtained from the National Centre for Cell Science (NCCS), Pune, India. The A431 skin cancer cells were cultured in Dulbecco’s Modified Eagle’s Medium (DMEM) supplemented with penicillin and 10% fetal bovine serum (FBS) as a monolayer in tissue culture Petri dishes. Cells were maintained at 37 °C in a humidified atmosphere of 5% CO_2_, and cell lines were maintained with regular passaging and the medium was replaced every 2 days. Cell viability was determined by the trypan blue dye exclusion method. 

#### 2.5.3. Biological Assays-Antiproliferation and Cytotoxicity Assays against Human Cancer Cell Lines 

##### Andrographolide (AG) Loading on Fe_3_O_4_/HAp NCs

Andrographolide (AG) was resuspended in dimethyl sulphoxide (DMSO), to give a stock concentration of 100 mM and was stored at −20 °C. DMSO was utilized as a vehicle control in all experiments involving andrographolide. Then 20 μM of AG was taken from the stock concentration and added into a 5 mL test tube. Then the optimum concentrations of a 50 μg/mL Fe_3_O_4_/HAp NCs suspension was added into 20 μM of the AG solution and incubated on the shaker at room temperature for 15 min. Afterwards, a permanent external magnet was used for separating the AG-loaded Fe_3_O_4_/HAp NCs and the sample was freeze dried at overnight. 

##### Antiproliferative Activity Assay

The MTT assay is used for cell viability and metabolic activity studies. This assay is based on the capability of a mitochondrial dehydrogenase enzyme from viable cells to cleave the tetrazolium rings of the pale yellow MTT (3-(4,5-dimethylthiazol-2-yl)-diphenyl tetrazolium bromide) which results in the formation of dark blue formazan crystals that are impermeable to cell membranes, thus resulting in its accumulation within healthy cells. The number of surviving cells is directly proportional to the level of the formazan product created. A431 cancer cells were seeded at a density of 1 × 10^4^ cells per well in 96-well plates. The cells were then treated with 100 μL of complete culture medium in the presence or absence of a series of increasing concentrations of test substances for 24 h to test the cytotoxicity on the A431 cells. Thereafter, the MTT solution in phosphate buffered saline (0.5 mg/mL) was added to each well and the microplate was incubated further for 4 h at 37 °C with the plates wrapped in aluminium foil in a humidified environment of 5% CO_2_. Finally, the crystals were solubilized by adding dimethyl sulfoxide (DMSO) solution. After complete solubilization of the purple formazan crystals formed by the live cells, the absorbance was read at 540 nm in a microplate reader. The results were analyzed using the average cytotoxicity data of the three independent experimental results and their associated errors. The inhibitory concentration (IC50), i.e., the drug concentration inhibiting 50% of the cell growth, was elucidated from the graph. Also, the same experimental procedure was adopted to determine the cytotoxicity effect of NCs using normal human epidermal keratinocyte HaCaT cells. 

##### Optical Microscopic Study to Detect Morphological Changes in A431 Cells

A431 cells were plated in 6 well sterile cell culture Petri dishes at 37 °C in a CO_2_ incubator and the cells were treated with 1 mL of complete culture medium containing nanocomposites at various concentrations for 24 h. The morphologies of the A431 cells were visualized using a phase contrast microscope. Untreated cells were retained as a control, and the morphological changes were visualized by the extent of cell roundedness.

##### Neutral Red Cytotoxicity Assay

The cytotoxicity was performed using the neutral red cytotoxicity assay, in which cells were plated in a 96-well microtiter plate, at a concentration of 1 × 10^4^ cells/well and incubated in a CO_2_ incubator at 37 °C to allow the cells to adhere. Then, the cells were treated with either drug (AG) or nanocomposites (Fe_3_O_4_/HAp NCs) or drug coated nanocomposites in three replicates. The plates were incubated for 24 h at 37 °C in a 5% CO_2_ incubator. DMSO was used as the drug control. No effect due to DMSO was observed. Then the old medium was substituted with fresh medium containing 50 μg/mL neutral red. The 96-well plates were placed in an incubator for 3 h. This allows the uptake of the dye into the lysosomes of viable and undamaged cells. Then the media was discarded and the cells were washed with 200 μL of neutral red washing solution. The dye was eluted from the cells by adding neutral red resorb solution (200 μL). The plate was further incubated for 30 min with rapid agitation on a microtiter plate shaker. The optical density (OD) of the eluted dye was read at 540 nm using a microplate reader. The experiments were conducted in triplicate. 

##### Assessment of Reactive Oxygen Species by DCFH-DA

A431 cells were seeded in 96-well plates, incubated for 24 h, and treated with either drug (AG) or nanocomposites (Fe_3_O_4_/HAp NCs) or drug coated nanocomposites for 24 h in DMEM-FBS medium. In order to detect the production of Reactive Oxygen Species (ROS), the cells were harvested, washed with PBS, re-suspended in PBS containing dichloro-dihydro-fluorescein diacetate (DCFH-DA) (10 mM), and incubated at 37 °C for 30 min. The ROS generated was measured by flow cytometry for green fluorescence at 530 nm.

##### Annexin V-FITC Apoptosis Assay 

The percentage of apoptotic cells was determined using an fluorescein isothiocyanate (FITC)-labeled annexin V (Annexin V-FITC) apoptosis detection kit according to the manufacturer’s instructions. An equal amount of cells per well were grown overnight in a 60 mm plate for 24 h and treated with either drug (AG) or nanocomposites (Fe_3_O_4_/HAp NCs) or drug coated nanocomposites for 24 h in DMEM-FBS medium. The cells were then grown for another 24 h. The percentage of apoptosis was quantified through flow cytometry using the FACScan Calibur (BD Biosciences, San Jose, CA, USA). The percentage of Annexin V positive cells were analyzed using Facs suite software (BD FACSVerse, BD Biosciences, San Jose, CA, USA).

## 3. Results and Discussion 

### 3.1. Physico-Chemical Properties Analysis 

**Figure 1:**
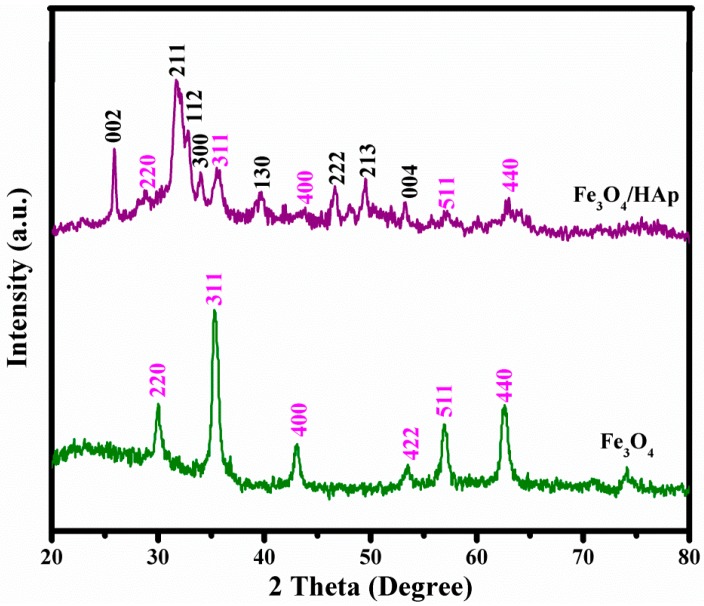
X-ray diffraction patterns of Fe_3_O_4_ nanoparticles and Fe_3_O_4_ nanoparticles on hydroxyapatite nanorod based nanostructures (Fe_3_O_4_/HAp) nanocomposites.

The structural and crystalline nature of the as-prepared Fe_3_O_4_ and Fe_3_O_4_/HAp nanocomposites were studied through X-ray diffraction analysis. Figure 1 shows the XRD patterns of the Fe_3_O_4_ nanoparticles; the peak position and relative intensity of the reflection peaks of the samples confirm the crystalline cubic spinel structure of the magnetite, which matched the standard XRD pattern of Fe_3_O_4_, JCPDS card. No (89-0691). The characteristic diffraction peaks can be identified at 2θ = 29.9°, 35.6°, 43.2°, 53.5°, 57.1°, 62.7°, 74.2°, and 75.1° which are marked by their miller indices (220), (311), (400), (422), (511), (440), (620), and (553), respectively, with the calculated lattice parameter *a* = 8.3891 Å.

From this X-ray diffraction analysis, the average particle size has been estimated by using the Scherrer formula:

$$D=\frac{0.9\;\lambda \;}{\beta \;\mathrm{COS}\theta }$$

where λ is the wavelength of the X-ray (0.1541 nm), β is the FWHM (full width at half maximum), θ is the diffraction angle, and *D* is the particle diameter size. The as-prepared samples exhibit highly crystalline XRD peaks with the average grain sizes of the Fe_3_O_4_ calculated to be 18 nm using the Debye-Scherer’s equation. The XRD pattern of the Fe_3_O_4_/HAp nanocomposites showed two different phases; the sequences of major typical peaks at 2θ = 25.66°, 31.39°, 32.09°, 32.49°, 39.62°, and 49.37° corresponding to the (002), (211), (112), (300), (202), (310), (222), (213), and (004) planes are crystal phases of HAp and 29.9°, 35.61°, 57.1°, and 62.71° corresponding to the (220), (311), (511), and (440) plans are crystal phases of Fe_3_O_4_, respectively. The crystal sizes were calculated to be 25 and 13 nm which corresponds to HAp and Fe_3_O_4_, respectively. These XRD results suggest the presence of a well crystallized cubic spinal phase of Fe_3_O_4_ and hexagonal phase HAp crystal structures were obtained and no other chemical impurities were detected.

**Figure 2:**
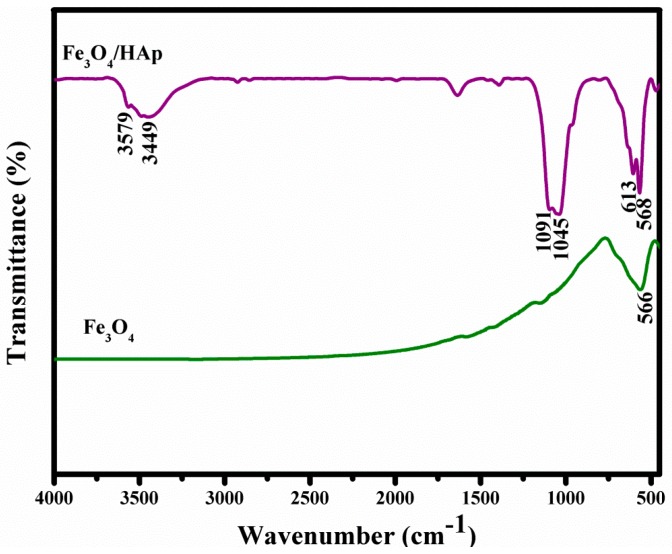
Fourier transform infrared spectroscopy (FTIR) spectra of Fe_3_O_4_ nanoparticles and Fe_3_O_4_/HAp nanocomposites.

The FTIR spectra of the Fe_3_O_4_ nanoparticles and Fe_3_O_4_/HAp nanocomposites are shown in Figure 2. The strong band at 566 cm^−1^ is characteristic of Fe–O vibration of the Fe_3_O_4_ nanoparticle. The typical adsorption bands at 508, 613, and 604 cm^−1^ corresponds to the bending vibration (ν_4_) of the phosphate groups. The absorption bands at 1045 and 1091 cm^−1^ are attributed to the stretching vibration (ν_3_) of the phosphate groups. The absorption peak at 3579 cm^−1^ corresponds to the stretching of the −OH bonds which confirms that the combination of Fe_3_O_4_ nanoparticles does not affect the OH site of the HAp. The broad absorption peaks around 3449 and 1637 cm^−1^ are assigned to the H_2_O of crystallization present in the as-prepared samples. 

**Figure 3:**
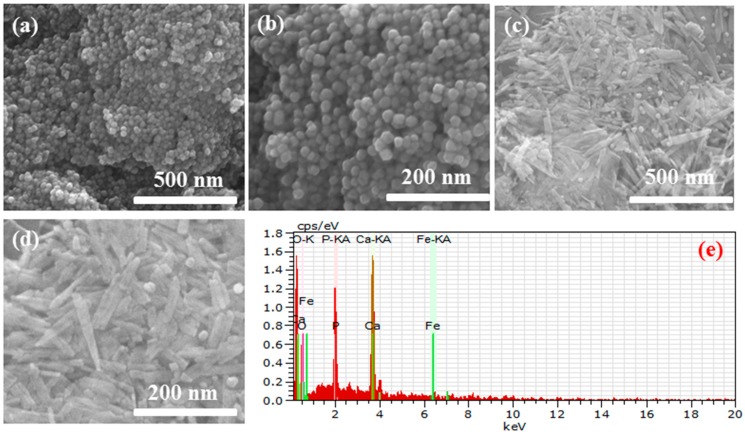
Field emission scanning electron microscopy (FESEM) images of Fe_3_O_4_ nanoparticles (**a**,**b**); Fe_3_O_4_/HAp nanocomposites (**c**,**d**) and (**e**) EDS spectrum of Fe_3_O_4_/HAp nanocomposites.

The morphology of the as-prepared Fe_3_O_4_ nanoparticles and Fe_3_O_4_/HAp nanocomposites were further investigated by field emission scanning electron microscopy (FESEM). Figure 3a,b shows the FESEM images of Fe_3_O_4_ nanoparticles and the sample has a homogeneously dispersed sphere like morphology with average particle sizes in the range of 20 nm. The typical FESEM images of the as-prepared nanocomposites are shown in Figure 3c,d. It shows that the Fe_3_O_4_ nanoparticles dispersed on the HAp nanostructures and the HAp has a rod-like morphology with a slightly needle shape. The size of the HAp nanorods in Figure 3c,d exhibits an average length and diameter in the range of 80 nm and 23 nm, respectively, and 18 nm sized Fe_3_O_4_ nanoparticles were homogeneously dispersed on the rod-like HAp nanostructures. The EDS spectrum (Figure 3e) of the Fe_3_O_4_/HAp nanocomposites shows that the sample consists of Ca, P, O, and Fe and no other impurities were detected. 

**Figure 4:**
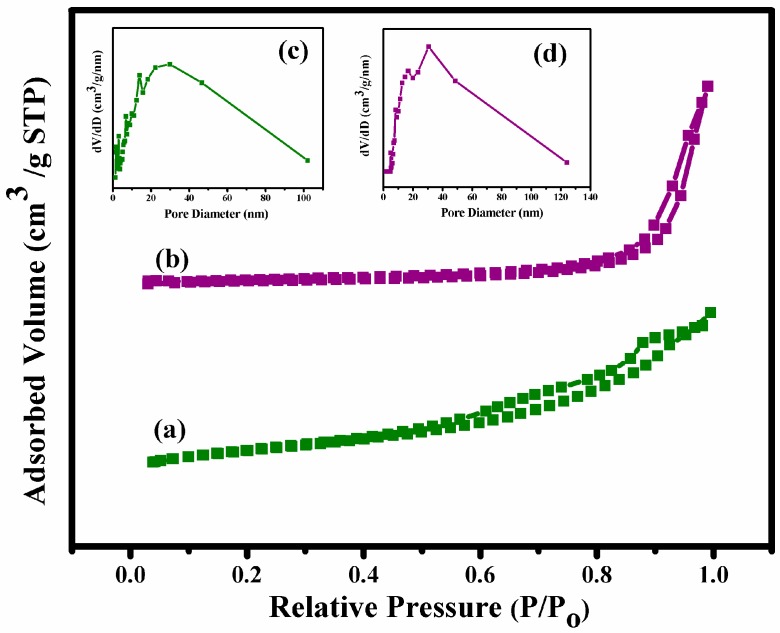
(**a**) Nitrogen (N_2_)-adsorption/desorption isotherms of Fe_3_O_4_ nanoparticles, and (**b**) Fe_3_O_4_/HAp nanocomposite. Inset Figure (**c**,**d**) shows the pore-size distribution of the Fe_3_O_4_ nanoparticles and Fe_3_O_4_/HAp nanocomposite, respectively.

The specific surface area (SSA) and pore size distribution of the Fe_3_O_4_ nanoparticles and Fe_3_O_4_/HAp nanocomposites were further evaluated by nitrogen adsorption-desorption analysis and the results were determined by a type IV isotherm loop at a relative pressure between 0 and 1. Figure 4 shows the BET specific surface area and Barrett-Joyner-Halenda (BJH) pore size distributions of the samples. Both samples showed mesoporous structures and the BJH calculations determined that the pore size distribution of both as-prepared samples in the range of 3 nm and 7–9 nm corresponds to the Fe_3_O_4_ nanoparticles and Fe_3_O_4_/HAp nanocomposites, respectively. The BET specific surface area of Fe_3_O_4_/HAp nanocomposites was observed to be 87 m^2^ g^−1^, which was higher than the Fe_3_O_4_ nanoparticles (40 m^2^ g^−1^). Therefore, the higher specific surface area of the Fe_3_O_4_/HAp nanocomposites is an excellent candidate for the high loading of drugs and controlled delivery biomedical applications.

**Figure 5:**
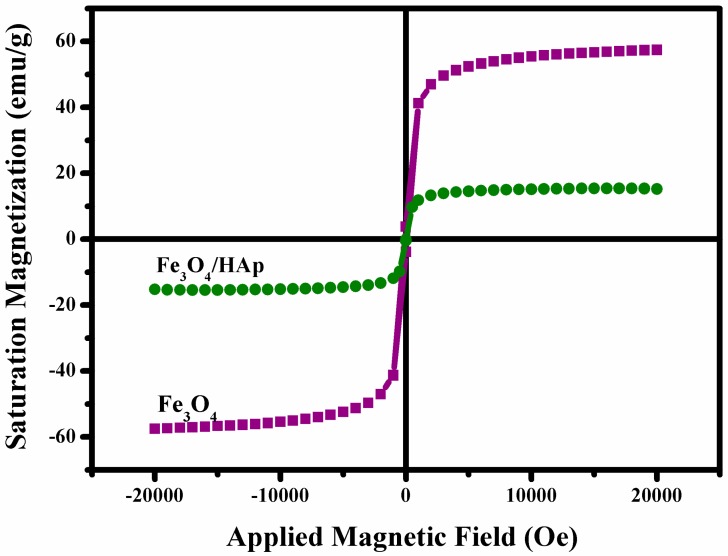
Magnetic (M-H) hysteresis loops of the samples: Fe_3_O_4_ nanoparticles and Fe_3_O_4_/HAp nanocomposites measured at room temperature.

The magnetic properties of the Fe_3_O_4_ nanoparticles and Fe_3_O_4_/HAp nanocomposites were investigated through vibrating sample magnetometry (VSM) at room temperature with an applied field of −2 KOe to 2 KOe, respectively. The hysteresis loop of both samples are shown in Figure 5 which exhibits a superparamagnetic nature at room temperature. The saturation magnetization (Ms) and coercivity (Hc) of the Fe_3_O_4_ nanoparticles are observed to be 60.5 emu/g and 0.05 Oe, respectively. A hysteresis measurement of the Fe_3_O_4_/HAp nanocomposites shows magnetization and coercivity around 18.2 emu/g and 0.09 Oe, respectively. The significance of this study is that the as-prepared Fe_3_O_4_/HAp nanocomposites have a better magnetic property and this is useful for targeted drug delivery with an assisting external magnetic field.

**Figure 6:**
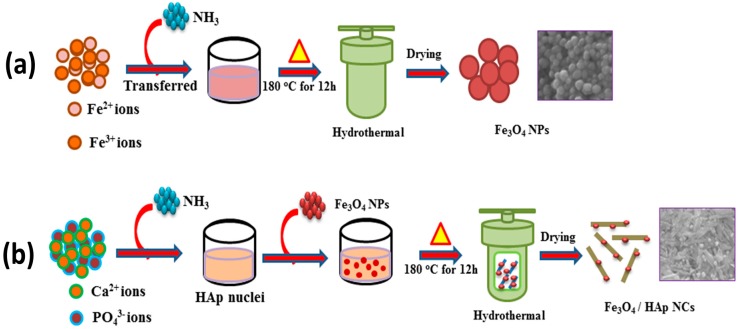
(**a**) Schematic illustrations for the synthesis of Fe_3_O_4_ NPs and (**b**) Fe_3_O_4_/HAp nanocomposites (NCs) synthesis through a hydrothermal process at 180 °C for 12 h.

Figure 6a shows the nucleation and growth mechanism of Fe_3_O_4_ NPs formed by a hydrothermal process at 180 °C for 12 h, which is the associated precipitation from ferric (Fe^2+^) and ferrous (Fe^3+^) iron in an alkaline aqueous solution (pH 10–12). The ferric (Fe^2+^) and ferrous (Fe^3+^) ions were hydrolyzed to form intermediate phases of Fe(OH)_2_ and Fe(OH)_3_ through ammonia water with a pH in the range of 10–12. Furthermore, the intermediate hydroxide phases were oxidized into the magnetite phase under the hydrothermal process at 180 °C for 12 h. The overall reaction is represented by Equation (1).

(1) $$2{\;\mathrm{Fe}}^{3+}+{\mathrm{Fe}}^{2+}+\;8{\mathrm{OH}}^{-}\to \;2\;\mathrm{Fe}{\left(\mathrm{OH}\right)}_{3}\;\mathrm{Fe}{\left(\mathrm{OH}\right)}_{2}\to {\;\mathrm{Fe}}_{3}O_{4}\;\left(s\right)+4H_{2}O$$

The schematic illustration in Figure 6b explains the formation mechanism of Fe_3_O_4_ nanoparticles dispersed on HAp nanorods. The precursors of CaCl_2_·2H_2_O and (NH_4_)_2_(HPO_4_) released Ca^2+^ and PO_4_^3−^ ions into the aqueous solutions and these ions reacted with ammonia water with a pH in the range of 10.5–11 to form an initial HAp nuclei. Then, the as-prepared Fe_3_O_4_ nanoparticles were added during the HAp nuclei formation. The hydrothermal temperature played an important role in improving the crystallinity of HAp and the orientational growth of rod-like formations, resulting in Fe_3_O_4_ nanoparticles that were dispersed on the HAp nanorods. The structural, morphological, and magnetic analyses were well supported for the nucleation growth mechanism of Fe_3_O_4_/HAp nanocomposites.

### 3.2. Biophysical Properties 

#### 3.2.1. In Vitro Cytotoxic Effect of Fe_3_O_4_/HAp Nanocomposites

**Figure 7:**
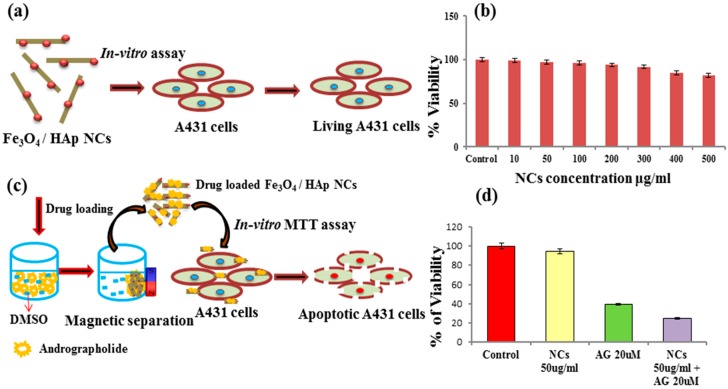
(**a**) Schematic illustrations for the loading of Fe_3_O_4_/HAp NCs on A431 cell lines todetermine the cytotoxicity of the samples; (**b**) In vitro biocompatibility and cell proliferation of the Fe_3_O_4_/HAp NCs by MTT assay with different dosages of 10 to 500 μg mL^−1^; (**c**) schematic diagram for andrographolide (20 μM) anti-cancer drug loading on Fe_3_O_4_/HAp NCs (50 μg mL^−1^) treated with the A431 cell lines and (**d**) In vitro biocompatibility and cell proliferation of the andrographolide-Fe_3_O_4_/HAp NCs by MTT assay.

The biomedical application of Fe_3_O_4_/HAp nanocomposites requires biocompatibility. Based on the literature available on toxicity and the increasing concern regarding the safety of graphene based nanocomposites, more conclusive examination is required. Cytotoxicity is an important factor for analyzing the biocompatibility of as-prepared Fe_3_O_4_/HAp nanocomposites. Figure 7a shows the mechanism of loading Fe_3_O_4_/HAp nanocomposites on A431 cell lines to determine the cytotoxicity of the samples. The cytotoxicity of the nanocomposite was investigated using the MTT assay in which the A431 cells were exposed to NC concentrations at 0–500 μg concentrations for 24 h. Figure 7b shows the MTT assay results which show the viability of cancer cells even at higher doses of Fe_3_O_4_/HAp nanocomposites, inferring the biocompatible nature of the nanoparticles. As the Fe_3_O_4_/HAp nanocomposites had no cytotoxic effect on the cancer cell line, it is likely that the nanocomposites would be non-toxic to normal cells as well. Previous reports are available on the dose dependent (50 to 300 mg mL^−1^) cytotoxicity effects of Fe_3_O_4_/HAp nanocomposites against human gastric carcinoma cells (MGC-803) [8]. This results demonstrates that the higher dosage of Fe_3_O_4_/HAp nanocomposites has no significant cytotoxic effect against MGC-803 cell lines. 

#### 3.2.2. Cytotoxic Effect of Andrographolide

The as-prepared Fe_3_O_4_/HAp NCs provide biocompatibility with high specific surface area (SSA) properties, and were utilized as drug nanocarriers toward the treatment of cancer. In this section, Andrographolide was used as an anti-cancer drug towards treating the cancer cells for cancer therapy applications. *Andrographis paniculata*, a traditional medicinal herb and Andrographolide (Andro), a diterpenoid lactone which is the major constituent, is used for treating a variety of diseases. Andrographolide extracted from the leaves of *Andrographis paniculata* has strong antiparasitic, antileishmanial, anti-inflammatory, anti-filaricidal, antimalaria, anticancer, and hepatoprotective properties [16,17,18]. The solubility of the compound plays a prominent role for the chemotherapeutic agent to be effective. Due to its diterpenoid lactone nature this compound has few limiting factors which actually make this drug as an effective target to kill cancer cells specifically. It is sparingly soluble in water and thus this nature restricts its bio distribution and localization. It is highly unstable in the extremes of gastrointestinal alkaline and acidic conditions. It has a very short biological half-life where it loses half of its pharmacologic activity and is thus just effective enough to kill cancer cells and also is difficult for cancer cells to develop resistance against this drug (t½ d is unstable) [19]. Furthermore, the drug is always exhibits some of side effects and these side effects are due to their lack of target specificity. Hence to overcome these limitations, a suitable delivery device within the desirable range for the diterpenes such as AG is required. Here, we have developed an Andro-Fe_3_O_4_/HAp nanocomposite based nanocarrier for the delivery of the hydrophobic plant bioactive AG. The negatively charged AG (zeta potential surface charge, −34.8 mV) [20] easily adsorbed on the positively charged Fe_3_O_4_/HAp NCs. Generally, the geometrical structures of Fe_3_O_4_/HAp NCs play a major role in the adsorbed anti-cancer drugs through electrostatic attractions. In particular, the crystal structure of HAp includes negatively charged PO_4_^3−^ and OH^−^ ions and positively charged Ca^2+^ ions lead to adsorption of the negatively charged drugs [21]. Moreover, the zeta potential charge of the pure Fe_3_O_4_ nanoparticles show a positive potential charge of 8 mV [22]. On the other hand, higher SSA of the Fe_3_O_4_/HAp NCs provides a better matrix for loading drugs. Previous studies reported that the as-prepared Fe_3_O_4_/HAp samples had high protein adsorption capacity (Qo) around 200.07 mg g^−1^ [8]. Based on our experimental results and several literature studies, the as-prepared Fe_3_O_4_/HAp NCs are a suitable nanocarrier for an anti-cancer drug delivery platform for cancer therapy. Figure 7c shows the schematic diagram for andrographolide (AG) adsorption onto Fe_3_O_4_/HAp nanocomposites. The 20 μM of AG was resuspended in dimethyl sulphoxide (DMSO) and the required amount of Fe_3_O_4_/HAp nanocomposite was added into the solution. The AG was completely adsorbed on the surfaces of the Fe_3_O_4_/HAp nanocomposite due to electrostatic attraction. After the adsorption, the AG loaded-Fe_3_O_4_/HAp nanocomposite was easily separated by applying an external magnetic filed. Afterwards, the A431 cells were treated with the AG loaded-Fe_3_O_4_/HAp nanocomposite and the apoptotic cells were determined through the MTT assay as shown in Figure 7d. Our previous reports explain that the results of the MTT assay showed the increase in the cytotoxic effect with the increasing dose of the andrographolide drug in the cancer cell lines [12]. We further proposed to check the role of the Fe_3_O_4_/HAp nanocomposite as a vehicle for targeting cancer cells. The A431 cells were treated with the vehicle control, Fe_3_O_4_/HAp NCs at a biocompatible concentration, andrographolide at a concentration less than IC50, and AG loaded Fe_3_O_4_/HAp NCs, and the cytotoxicity was checked after 24 h. Figure 7d shows the increase in toxic effects in the cancer cells when treated with the Fe_3_O_4_/HAp nanocomposite with andrographolide, which infers that the Fe_3_O_4_/HAp nanocomposite has a high adsorption capacity that results in the potential application of nanoparticles as a better vehicle for anticancer drugs to treat cancer. When andrographolide was loaded onto the Fe_3_O_4_/HAp nanocomposite, the selectivity of andrographolide increased and was used for developing andrographolide as a therapeutic agent for cancer therapy. This is also low cost and is capable of ferrying anticancer drugs into malignant cells while sparing healthy cells. This also leads to the reduction of the dose required because the drug is delivered directly to target cells. This magnetic carrying platform involves the use of drug loaded Fe_3_O_4_/HAp nanocomposites that are transported through the bloodstream to the targeted site by an external magnetic field. 

#### 3.2.3. Optical Microscopy Study

**Figure 8:**
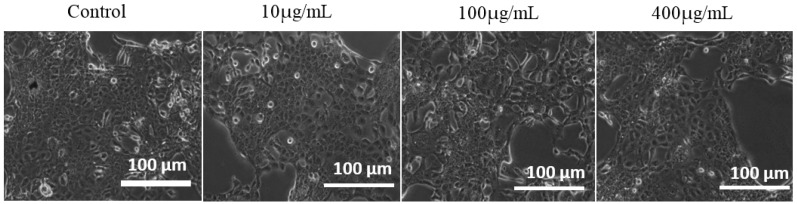
Light microscopy images of A431 cells treated with different dosages of the Fe_3_O_4_/HAp nanocomposite.

The A431 cancer cells were plated in 6 well plates. After 24 h, the cells were treated with 1 mL of complete culture medium containing Fe_3_O_4_/HAp nanocomposites at various concentrations for 24 h. The untreated control cells and the nanoparticle treated cells were observed under a phase contrast microscope to detect any morphological changes that occurred, and the images are presented in Figure 8. Morphological changes such as roundedness and irregular cell shapes were analysed but the treated cells exhibited no changes in morphology. Hence the as-prepared nanocomposites are nontoxic to the cancer cells and this result was in good agreement with the MTT assay as shown in Figure 7b. 

#### 3.2.4. Cytotoxicity Effect of Fe_3_O_4_/HAp NCs on Normal Human Epidermal Keratinocyte HaCaT Cells

**Figure 9:**
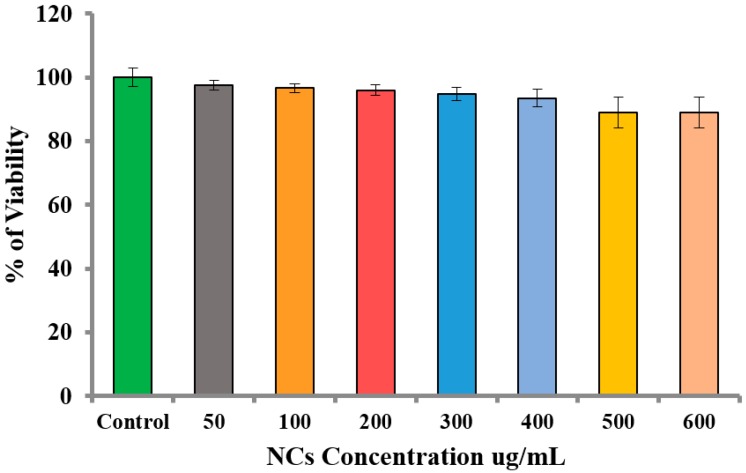
Dose-dependent toxicity of Fe_3_O_4_/HAp NCs in HaCaT cells. Cells were treated with 50, 100, 200, 300, 400, 500, and 600 μg/mL of Fe_3_O_4_/HAp NCs for 24 h. The cell viability was measured by MTT assay in triplicate.

The normal human epidermal keratinocyte HaCaT cells were used to determine the cytotoxicity effect of the synthesized Fe_3_O_4_/HAp NCs. Figure 9 shows the viability (%) of HaCaT cells exposed to the control, 50, 100, 200, 300, 400, 500, and 600 μg/mL of Fe_3_O_4_/HAp NCs at 24 h. The MTT-assay data are expressed as the mean ± standard deviation of three independent assays in triplicate. The cell viability was not significantly decreased even when the Fe_3_O_4_/HAp NCs concentration was up to 400 μg/mL and the cell viability only slightly decreased as a function of the dosage level increase to 600 μg/mL. This result indicated excellent biocompatibility of the synthesized Fe_3_O_4_/HAp NCs, inferring that they are good candidates for drug delivery nanocarriers. 

#### 3.2.5. Neutral Red Cytotoxicity Assay

**Figure 10:**
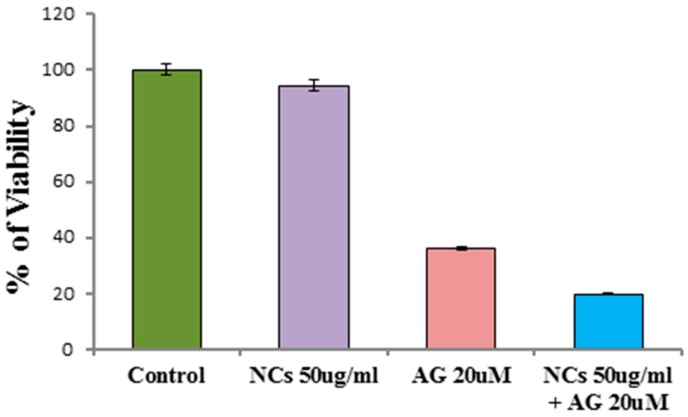
Neutral red cytotoxicity assay of the control, Fe_3_O_4_/HAp NCs, drug (AG), and AG-loaded Fe_3_O_4_/HAp NCs.

The colorimetric assay was used for the quantification of the membrane permeability and lysosomal activity of the cells in response to the desired substance. The neutral red (NR) assay procedure is a cell survival/viability assay based on the ability of viable cells to incorporate and bind neutral red within the lysosomes of adherent cells. NR is a weak cationic dye that readily penetrates the cell membrane and accumulates intracellularly in lysosomes (lysosomal pH < cytoplasmic pH), where it binds with anionic sites to the lysosomal matrix. Figure 10 shows the neutral red cytotoxicity assay of the control, Fe_3_O_4_/HAp NCs, drug (AG), and AG-loaded Fe_3_O_4_/HAp NCs in three replicates. The cytotoxic effect of the drug caused changes in the cell surface or the sensitive lysosomal membrane lead to lysosomal fragility and other irreversible changes. These alterations caused by the action of the drug results in a decreased uptake and binding of NR. Hence it is easy to distinguish between viable, damaged, or dead cells, which are the basis of the assay. The quantity of dye incorporated into cells is measured by spectrometry at 540 nm, and is directly proportional to the number of cells with an intact membrane. Drug loaded Fe_3_O_4_/HAp nanocomposites showed higher cytotoxicity and lesser intact lysosomes when compared to the control cells.

#### 3.2.6. Measurement of ROS Production

**Figure 11:**
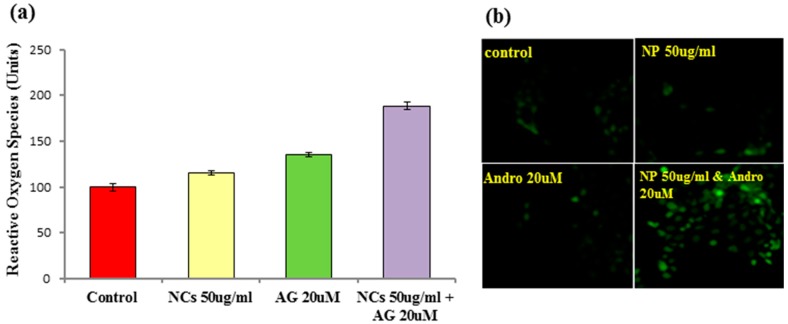
(**a**) Measurement of ROS production of the control, NCs, andrographolide (AG), and AG loaded on Fe_3_O_4_/HAp nanocomposites in A431 cells and (**b**) Fluorescent images of A431 cells treated with different dosages of AG loaded on Fe_3_O_4_/HAp nanocomposites.

Cancer cells show increased oxidative stress compared to normal cells, which is associated with the increased generation of reactive oxygen species (ROS) and alterations in metabolic activity. Our studies show that ROS generation is due to the exhaustion of antioxidant defense systems and the impairment of lysosomal integrity. This generates increased ROS, causing the loss of mitochondrial membrane potential and mediating mitochondrial damage and apoptosis. Apoptosis is the highly organized process of programmed cell death characterized by a set of morphological changes and biochemical steps, including the translocation of phosphatidylserine from the inner to the outer layer of the plasma membrane and the formation of apoptotic bodies as a result of chromatin condensation and fragmentation of the cell. Hence Fe_3_O_4_/HAp nanocomposites were efficient drug carriers and were able to trigger apoptosis in A431 skin cancer cells. Figure 11a shows the measurement of ROS production of andrographolide loaded on the Fe_3_O_4_/HAp nanocomposite in A431 cells. The involvement of ROS such as H_2_O_2_, O_2_, and peroxynitrite induced by andrographolide delivered by Fe_3_O_4_/HAp nanocomposites in the apoptotic pathway was studied using the dye 2, 7-dichlorodihydrofluorescein diacetate (DCFH-DA), which shows enhanced fluorescence when oxidative stress is generated intracellular. The fluorescence generated due to the hydrolysis of DCFH-DA to dichlorodihydrofluorescein (DCFH) by non-specific cellular esterases and the subsequent oxidation of DCFH by peroxides was measured by flow cytometry for green fluorescence at 530 nm. The intensity of ROS production was significantly high when the cells were incubated with the drug loaded Fe_3_O_4_/HAp nanocomposites compared to the control group. The fluorescent images were taken using a fluorescent microscope. Figure 11b shows the fluorescent images of the A431 cells treated with different dosages of andrographolide loaded on Fe_3_O_4_/HAp. The results showed that the drug loaded Fe_3_O_4_/HAp nanocomposites were able to generate ROS, and apoptotic cell death in A431 cells is mediated by ROS signaling.

#### 3.2.7. Detection of Apoptosis Using Annexin-V-FITC and PI Dual Staining

**Figure 12:**
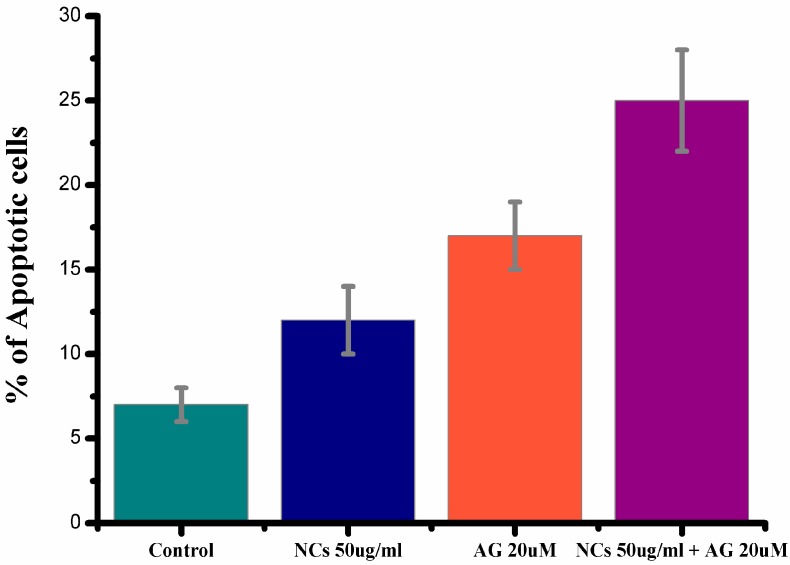
Annexin V-FITC/PI double staining analysis of apoptosis in A431 cells (24 h) and percentages of apoptosis in A431 cells.

The treatment of A431 skin cancer cells with the AG loaded Fe_3_O_4_/HAp nanocomposites induced apoptosis. The cell death induced by andrographolide delivered using the Fe_3_O_4_/HAp nanocomposites was measured by flow cytometry using the annexin V-FITC/PI staining method. The cells were seeded in 60 mm culture dishes and treated with either AGor Fe_3_O_4_/HAp nanocomposites or AG loaded Fe_3_O_4_/HAp nanocomposites in the DMEM FBS medium. The cells entered the apoptotic stage, and some cells were necrotic. The cells were then incubated with the recommended concentrations of annexin V-FITC with and without PI. The Annexin V-FITC conjugate, which is a fluorescent probe, binds to cells in early apoptosis, where phosphatidylserine is translocated to the external portion of the membrane and quantified through flow cytometry using the FACScan Calibur. The percentage of Annexin V positive cells was analyzed using the Facs suite software. Figure 12 demonstrates the Annexin V-FITC/PI double staining analysis of apoptosis in the A431 cells (24 h) and the percentages of apoptosis in the A431 cells. As can be seen, the AG loaded Fe_3_O_4_/HAp nanocomposites shows higher apoptotic cells than the control, Fe_3_O_4_/Hap, and AG. 

In the present studies, and in particular the physico-chemical and bio-physical properties of the dual crystal phases, geometrical properties, higher magnetic property, large specific surface area, higher drug loading capacity, and biocompatibility infers that the Fe_3_O_4_/HAp nanocomposites used as nanodrug carriers are potential candidates for cancer therapy applications. 

## 4. Conclusions

In summary, we successfully developed a drug nanocarrier made of the mesoporous Fe_3_O_4_/HAp nanocomposite through a hydrothermal technique at 180 °C for 12 h, and detailed investigations demonstrated that its low pH level controlled the drug release behavior. The structural, chemical, morphological, and magnetic properties of the samples were studied through various analytical measurements. Various experimental results demonstrated that the as-prepared sample consists of dual crystal phases including the cubic crystal structure of magnetite and the hexagonal structure of Hap, and no other mixed phases were present in the samples. In biological application, the as-prepared samples show a much lower in vitro cytotoxicity effect on the A431 cell lines; therefore, the presented nanocomposites show excellent biocompatability. Also, andrographolide loaded on the Fe_3_O_4_/HAp nanocomposite shows high antiproliferative activities and the induction of apoptosis against the A431 cell lines with rapid time. Therefore, this presented Fe_3_O_4_/HAp nanocomposite could decrease drug delivery time and dosage, increase drug loading capacity, and reduce the suffering of cancer patients, which is what the magnetic based targeted drug delivery system entails.
